# Microbiome insights from a South African cultural and natural landmark cave using metagenomics next-generation sequencing

**DOI:** 10.1128/mra.01183-24

**Published:** 2025-02-18

**Authors:** Olubukola Oluranti Babalola, Afeez Adesina Adedayo, Saheed Adekunle Akinola

**Affiliations:** 1 Food Security and Safety Focus Area, Faculty of Natural and Agricultural Sciences, North-West University, Mmabatho, South Africa; 2 Department of Microbiology and Parasitology, School of Medicine and Pharmacy, College of Medicine and Health Sciences, University of Rwanda, Butare, Rwanda; Montana State University, Bozeman, Montana, USA

**Keywords:** microbial biotechnology, cave soil microbiome, one health, environmental safety, agro-genomics, anthropogenic interference

## Abstract

The microbiome study of Sterkfontein Cave (a natural and cultural cave) revealed fascinating insights into its metagenome study and functional annotation. The largely unexplored cave soil microbiota showcases intricate survival adaptations with promising potential for various human applications. Here, we report the microbial diversity and functions associated with Sterkfontein Cave soil.

## ANNOUNCEMENT

During the 1800s, the Sterkfontein Cave gained popularity as a tourist hotspot, attracting both amateur enthusiasts and seasoned researchers. Despite this attention, little effort has been made to evaluate the abundance of microorganisms thriving within the inner recesses of stone walls and speleothems. Understanding the metagenome of Sterkfontein Caves could provide insights to address the question of “The function of our microbiota: who is out there and what do they do?” Cave-dwelling organisms exhibit complex survival adaptations, with cave bacteria producing secondary metabolites that have antimicrobial, anti-inflammatory, and anticancer potential (1). This study aimed to report the complexity of Sterkfontein Cave soil microbiome and functional attributes of medical and agricultural importance.

In November‒December 2022, soil samples from Sterkfontein Cave located near the city of Mogale, Gauteng (26^o^0'57.024" S 27^o^44'9.6" E) were collected in triplicates from three different locations inside the core dark zone of the cave (SK), about 20–50 m from the cave entrance and also from the lawn area (surrounding soil close to the cave) of the cave (SKC) to serve as a control (2). Thirty (30 g) grams of soil samples were collected from the first layer of the soil using a sterile scalpel and transported to the laboratory in a cooler box filled with ice and then stored at ‒20°C for a week (3, 4). DNA extraction from each 5 g soil sample was conducted using the QIAGEN DNeasy PowerMax soil kit (DNeasy MOBIO, Carlsbad, CA) following the manufacturer’s instructions. The DNA concentration was evaluated by fluorescence using the Quant-iT PicoGreen dsDNA kit (Invitrogen, Carlsbad, CA, USA) and a DQ 300 fluorometer (Hoefer Scientific Instruments, California). Afterward, libraries were generated using the Nextera DNA Flex library preparation kit (Bio-Active Co., Ltd). Twenty to 50 ng DNA was used to prepare the libraries. The samples underwent fragmentation and the addition of adapter sequences. The final concentrations of the libraries were measured using the Qubit double-stranded DNA (dsDNA) HS assay kit (Life Technologies), and the average DNA fragment lengths were determined using a 2100 Bioanalyzer (Agilent Technologies). The insert library size ranges between 300-900 bp (average 500 bp). The libraries underwent 2 × 150 bp sequencing on the NovaSeq 6000. The subsequent analysis of the reads was conducted using the default parameters of the Metagenomic Rapid Annotations using Subsystems Technology (MG-RAST) server v4.0.3 (Table 1). Within the MG-RAST server, quality control for raw reads was performed using SolexaQA (v2.5) to trim low-quality reads (5). The evaluation of sample sequencing error, relying on artificial duplicate read measurements, was achieved through duplicate read inferred sequencing error estimation (DRISEE v1.2) (6). Furthermore, the pipeline uses the Bowtie2 aligner to screen the reads for undesired genomes linked to model organisms viz. mice, humans, cows, and other animals (7). Within the same pipeline, the BLAST-like alignment tool (BLAT) algorithm was employed to annotate the sequences (8) against the M5NR (–v2.0) database (9).

**TABLE 1:** Basic analytical statistics, quality information, and project accession numbers from NCBI and MG-RAST databases

Sample	No. of raw sequence reads	NCBI BioProject number	Accession number of triplicate samples	No. of reads that passed quality control	No. of known proteins predicted	Mean read values (Domain in %)	Mean read values (Bacterial phyla in %)
SK (Sterkfontein Cave soil)	SK1‒25,579,360, SK3‒19,730,081, SK4‒23,852,012	PRJNA982608	SK1‒ SRX20716463 SK3‒ SRX20716464 SK4‒ SRX20716465	SK1‒4,583,206, SK3‒1,545,288, SK4‒1,781,749	SK1‒1,333,622, SK3‒407,454, SK4‒931,962	Bacteria ‒ 98.66 Archaea ‒ 0.05 Eukaryota ‒1.20 Viruses ‒0.07, unclassified sequences‒0.02	Proteobacteria 81.74, Actinobacteria‒10.71, Bacteroidetes‒10.57, Planctomycetes‒0.16, Acidobacteria ‒0.20, Firmicutes‒1.03
SKC (Surrounding/ Lawn soil)	SKC‒24,137,281, SKC3‒11,005,640, SKC4‒9,483,602		SKC‒ SRX20716466 SKC3‒ SRX20716467 SKC4‒ SRX20716468	SKC‒1,195,433, SKC3‒1,007,411, SKC4‒1,195,433	SKC‒1,174,881, SKC3‒989,441, SKC4‒1,174,881	Bacteria ‒ 98.11 Archaea ‒ 0.44 Eukaryota ‒1.29 Viruses ‒0.03 Unclassified sequence ‒ 0.13	Proteobacteria‒49.99, Actinobacteria‒25.07, Bacteroidetes‒7.14, Planctomycetes‒3.26, Acidobacteria‒3.09 Verrucomicrobia‒2.41

The domain distribution of microbial taxa showed mean read values assigned to Bacteria, Archaea, Eukaryota, viruses, and unclassified sequences for both Sterkfontein cave and lawn soil samples, respectively. Bacteria phyla such as *Proteobacteria* and *Actinobacteria* were the most abundant, and other phyla *viz. Bacteroidetes*, *Planctomycetes*, and *Firmicutes* were conspicuously high in the cave sample, whereas *Acidobacteria*, *Planctomycetes*, and *Verrucomicrobia* were significant in the lawn sample (Table 1).

Functional annotation based on subsystem analysis indicated that clustering based on subsystems comprised 13.44%, whereas amino acids and derivatives accounted for 11.41%. Carbohydrates sequences constituted 9.55%, along with other beneficial functional traits crucial for plant growth and health.

## Data Availability

The metagenomes of cave soil sequence reads were submitted to the NCBI with BioProject number PRJNA982608 and triplicate’s Sequence Read Archive (SRA) accession numbers SK1‒ SRX20716463, SK3‒ SRX20716464, and SK4‒ SRX20716465 (Sterkfontein Cave), SKC‒ SRX20716466, SKC3‒ SRX20716467, and SKC4‒ SRX20716468 (Lawn/bulk soil).
